# Unaltered intravenous prion disease pathogenesis in the temporary absence of marginal zone B cells

**DOI:** 10.1038/s41598-019-55772-w

**Published:** 2019-12-13

**Authors:** Barry M. Bradford, Neil A. Mabbott

**Affiliations:** The Roslin Institute & Royal (Dick) School of Veterinary Sciences, University of Edinburgh, Easter Bush, EH25 9RG UK

**Keywords:** Neuroimmunology, Infectious diseases, Infection

## Abstract

Prion diseases are a unique, infectious, neurodegenerative disorders that can affect animals and humans. Data from mouse transmissions show that efficient infection of the host after intravenous (IV) prion exposure is dependent upon the early accumulation and amplification of the prions on stromal follicular dendritic cells (FDC) in the B cell follicles. How infectious prions are initially conveyed from the blood-stream to the FDC in the spleen is uncertain. Addressing this issue is important as susceptibility to peripheral prion infections can be reduced by treatments that prevent the early accumulation of prions upon FDC. The marginal zone (MZ) in the spleen contains specialized subsets of B cells and macrophages that are positioned to continuously monitor the blood-stream and remove pathogens, toxins and apoptotic cells. The continual shuttling of MZ B cells between the MZ and the B-cell follicle enables them to efficiently capture and deliver blood-borne antigens and antigen-containing immune complexes to splenic FDC. We tested the hypothesis that MZ B cells also play a role in the initial shuttling of prions from the blood-stream to FDC. MZ B cells were temporarily depleted from the MZ by antibody-mediated blocking of integrin function. We show that depletion of MZ B cells around the time of IV prion exposure did not affect the early accumulation of blood-borne prions upon splenic FDC or reduce susceptibility to IV prion infection. In conclusion, our data suggest that the initial delivery of blood-borne prions to FDC in the spleen occurs independently of MZ B cells.

## Introduction

Prion diseases (also known as transmissible spongiform encephalopathies) are unique, infectious, invariable fatal, neurodegenerative diseases. Infectious prion particles appear to be comprised almost entirely of PrP^Sc^ (misfolded forms of the host’s cellular prion protein, PrP^C^)^1^ suggesting that prions are infectious proteins^2,3^. The accumulation of PrP^Sc^ in the brain during prion disease coincides with extensive astrocytic and microglial activation and ultimately leads to neurodegeneration coincident with a spongiform (sponge-like) pathology (vacuolation) in the brain.

Infection with some natural prion diseases including natural sheep scrapie and chronic wasting disease in cervids occurs after peripheral exposure to infectious prions, for example by oral ingestion of food or pasture contaminated with prions. Bovine spongiform encephalopathy in cattle can be zoonotic, as the consumption of contaminated food in the UK led to the emergence of a novel human prion strain known as variant Creutzfeldt-Jakob disease (vCJD). After peripheral infection some prion agent strains accumulate and replicate first upon stromal follicular dendritic cells (FDC) in the B-cell follicles of host’s secondary lymphoid tissues including the Peyer’s patches, lymph nodes and spleen (reviewed in^4^). After their amplification within these tissues the prions then infect the sympathetic and parasympathetic nervous systems in order to establish CNS infection^5–8^. Studies in mice suggest that after peripheral exposure the initial replication of certain prion agents within the host’s secondary lymphoid tissues is important to efficiently establish CNS infection^5,6,9–12^.

The marginal zone (MZ) in the spleen provides a border around the lymphocyte-containing white pulp region and contains a marginal sinus through which the peripheral blood percolates as it travels towards the red pulp^13^. Specialized subsets of B cells and macrophages are positioned within the MZ to continuously monitor the blood-stream and remove pathogens, toxins and apoptotic cells. The MZ B cells, for example, capture blood-borne antigens and antigen-containing immune complexes^13–16^. By continually shuttling between the MZ and the B-cell follicle these cells efficiently capture and deliver systemic antigens and antigen-containing immune complexes to the FDC in the B cell follicles^14,15^. The innate-like characteristics of the MZ B cells also enable them to initiate rapid T-cell-independent antibody responses to certain polysaccharide antigens such as those from encapsulated bacteria^17,18^.

In the UK a small number of accidental iatrogenic cases of the human prion disease vCJD have been described in patients that received blood or blood products prepared from donors infected with vCJD prions^4,19–21^. After intravenous (IV) injection with prions their early amplification upon splenic FDC is also essential for efficient disease pathogenesis^12^. However, little is known of the cellular and molecular processes that mediate the initial propagation of blood-borne prions towards the FDC within the spleen. Prions can be captured and acquired by FDC as prion-containing complement component-opsonized complexes^22–27^. Furthermore, aged mice have a significantly reduced susceptibility to IV prion injection, and this coincides with disturbances to the cellular organization within the MZ that impede the transport of antigen-containing immune complexes and prions to FDC^12^. Therefore, since MZ B cells capture and deliver blood-derived antigens and antigen-containing immune complexes to splenic FDC, we tested the hypothesis that MZ B cells similarly shuttle blood-borne prions towards FDC^12^. Specifically, we determined whether the depletion of MZ B cells from the MZ niche by blocking integrin function^28^ would impede the initial delivery of prions from the blood-stream to FDC, and by doing so, reduce susceptibility to IV prion infection.

## Results

### Treatment with anti-LFA-1 and anti-α4 displaces MZ B cells from the splenic MZ

**Figure 1:**
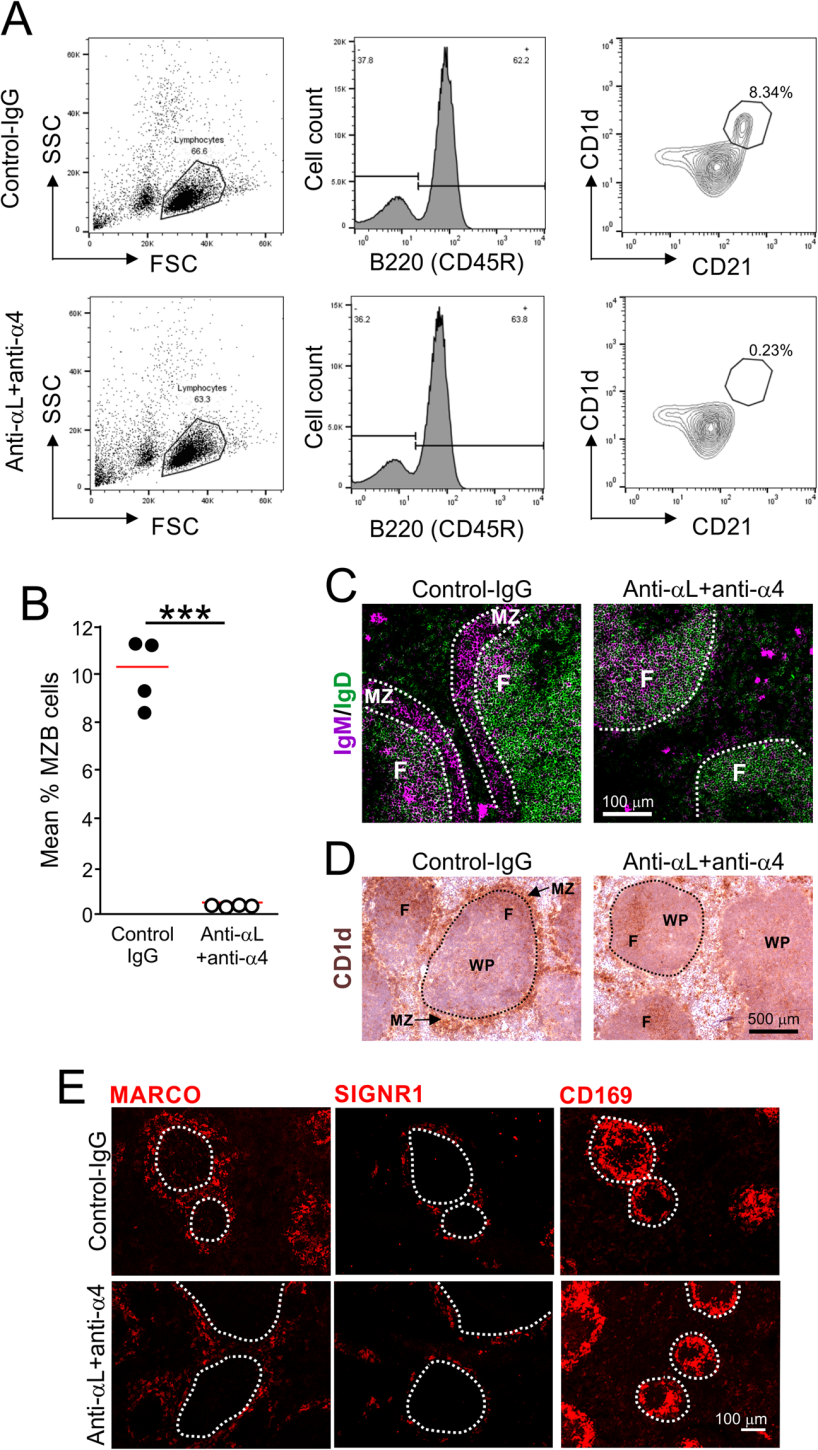
Displacement of MZ B cells from the splenic MZ by antibody-mediated blockade of the integrins LFA-1 (αLβ2) and α4β1. Mice were treated with anti-αL and anti-α4 mAb (anti-αL + anti-α4), or isotype-matched control-Ig (*n* = 4 mice/group), and spleens analysed seven days later. (**A**) Gating strategy used to identify B220 + CD21^hi^CD1d^hi^ MZ B cells by flow cytometry. (**B**) The percentage of B cells that were B220 + CD21^hi^CD1d^hi^ MZ B cells was significantly reduced in the spleens of anti-αL + anti-α4 treated mice. ***P < 0.001, Student’s t-test; *n* = 4 mice/group; closed circles, control-Ig; open circles, anti-αL + anti-α4; horizontal bars, median. (**C**) Representative immunostaining of IgM + (violet) and IgD + (green) cells in the spleens of mice from each treatment group. This analysis showed that IgM + MZ B cells were absent from the MZ region of the spleens of anti-αL + anti-α4 treated mice. MZ, marginal zone; F, B-cell follicle; broken line shows the boundary of the MZ; scale bar, 100 µm. (**D**) Representative immunostaining of CD1d + (brown) cells in the spleens of mice from each treatment group. Haematoxylin (blue) was used as a nuclear counterstain. This analysis showed that CD1d + MZ B cells were absent from the MZ region of the spleens of anti-αL + anti-α4 treated mice. WP, white pulp region; broken line shows the boundary of the MZ; scale bar, 500 µm. (**E**) Representative immunostaining shows a similar distribution of MARCO + (left-hand column), SIGNR1 + (middle column) and CD169 + (right-hand column) macrophages in the spleens of mice from each treatment group. Broken line shows the relative positioning of the marginal zinus. Scale bar, 100 µm.

Mouse MZ B cells express the integrins LFA-1 (lymphocyte function-associated antigen 1; αLβ2) and α4β1 highly and this enables them to bind to the adhesion molecules ICAM-1 (intracellular adhesion molecule 1) and VCAM-1 (vascular cell adhesion molecule 1) that are expressed in the MZ by the marginal sinus lining cells^28^. Antibody-mediated inhibition of this binding rapidly releases and displaces the MZ B cells from the MZ^28^. Here, the temporary displacement of MZ B cells from the splenic MZ was achieved as described previously^28–30^ by IV treatment of mice with anti-αL and anti-α4 monoclonal antibodies (mAb) to specifically inhibit the LFA-1 (αLβ2) and α4β1 integrins (termed anti-αL + anti-α4, herein after). Some mice were injected IV with non-specific Ig isotype-matched antibodies as a control (control-Ig). Spleens were analysed 7 days after treatment. Within the splenic B lymphocyte compartment mouse MZ B cells can be identified by flow cytometry as B220 + CD21^hi^CD1d^hi^ cells^13,28^. Here, flow cytometry confirmed that anti-αL + anti-α4 treatment led to a highly significant reduction in the abundance of MZ B cells in the spleen (Fig. 1A,B).

By immunohistochemistry (IHC) MZ B cells can be readily identified within the splenic MZ based on their high expression of IgM and low expression of IgD, or their high expression of CD1d^12,28^ (Fig. 1C,D). Our IHC analysis confirmed an almost complete absence of IgM + and CD1d + MZ B cells from the MZ of anti-αL + anti-α4 treated mice (Fig. 1C,D). Together these data confirm that MZ B cells are displaced from the splenic MZ by treatment with anti-αL + anti α4 mAb.

The MZ also contains specialized populations of macrophages that are situated within two separate continuous layers on either side of the marginal sinus. The MZ macrophages form a ring within the outer layer of the MZ and express the macrophage receptor with collagenous structure (MARCO) scavenger receptor. Some of these MZ macrophages also express the C-type lectin specific intracellular adhesion molecule-grabbing non-integrin receptor 1 (SIGNR1). The inner layer of MZ metallophilic macrophages express CD169 (siglec-acid-binding immunoglobulin-like lectin 1; SIGLEC1; Fig. 1E, upper panels). Our IHC analysis indicated that the distribution of these macrophage populations was not affected in the spleens of anti-αL + anti-α4 treated mice (Fig. 1E, lower panels), in agreement with previously published data^28^.

### Temporary displacement of MZ B cells does not affect the early accumulation of PrP^Sc^ in the spleen

**Figure 2:**
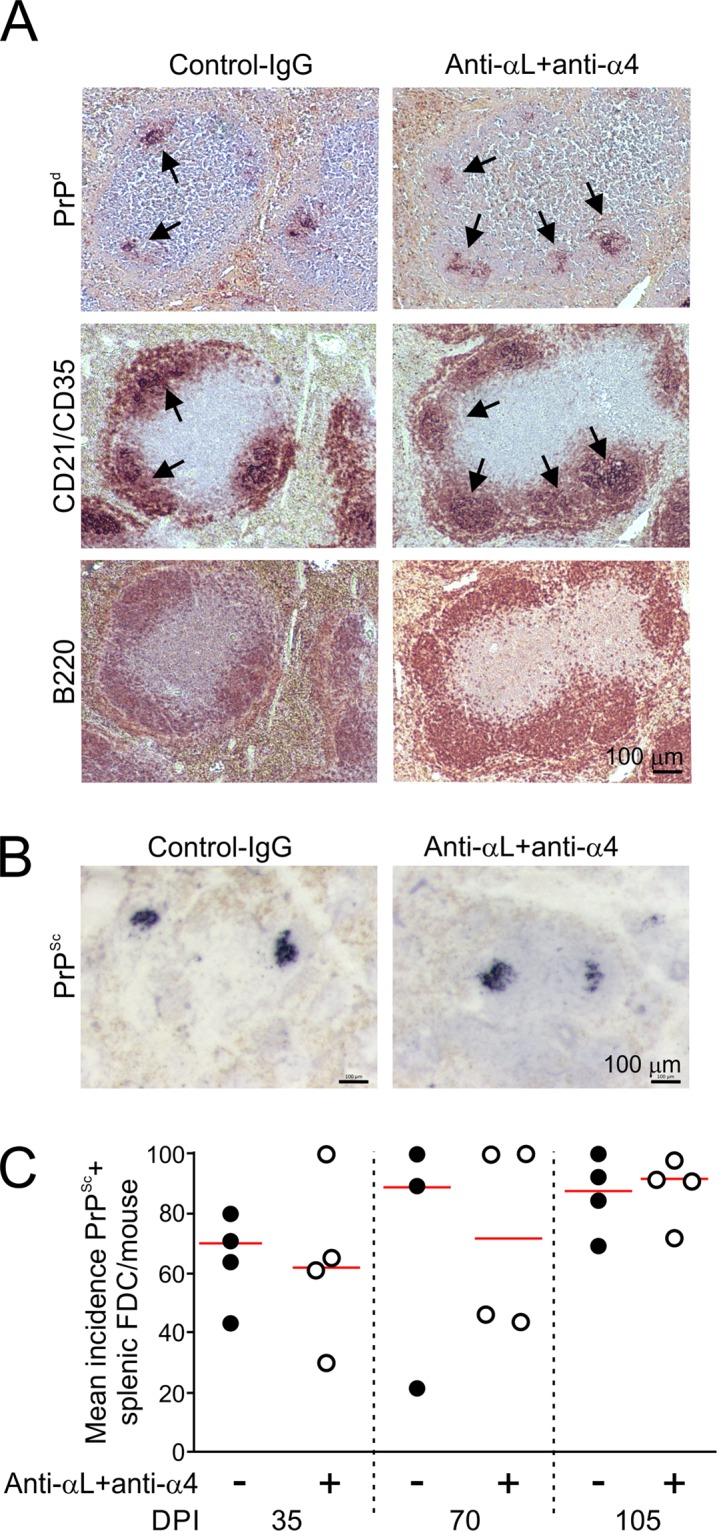
Temporary displacement of MZ B cells does not affect the early accumulation of PrP^Sc^ upon FDC in the spleen. Mice were treated with anti-αL and anti-α4 mAb (anti-αL + anti-α4), or isotype-matched control-Ig (*n* = 4 mice/group), and seven days later injected IV with ME7 scrapie prions. Spleens were analysed at 35, 70 and 105 days after IV prion exposure. (**A**) Immunohistohemical (IHC) analysis shows heavy prion disease-specific PrP^d^ accumulations (upper row) were detected in association with FDC (CD21/CD35 + cells, arrows; middle row) in the B cell follicles (B220 + cells, bottom row) of mice from each treatment group. Haematoxylin (blue) was used as a nuclear counterstain. Scale bar, 100 µm. (**B**) Paraffin-embedded tissue immunoblotting was used to confirmed the presence of prion-specific, relatively PK-resistant, PrP^Sc^ (blue/black, arrows). Scale bar, 100 µm. (**C**) Similar frequencies of PrP^Sc^ + FDC were detected in the spleens of mice from each treatment group. Closed circles, control-Ig; open circles, anti-αL + anti-α4; horizontal bars, median. *n* = 4 mice/group.

Next, we assessed whether the depletion of MZ B cells would affect the early accumulation of prions upon splenic FDC after IV exposure. Mice were injected with anti-αL + anti-α4 mAb (or control-Ig) as above, and seven days layer injected IV with a low (0.1%) dose of ME7 scrapie prions. As anticipated, by 35 days after IV injection with prions, heavy prion disease-specific PrP (PrP^d^) accumulations were detected by IHC in association with FDC in the spleens of control-Ig treated mice (Fig. 2A). Paraffin-embedded tissue immunoblot analysis demonstrated the presence of prion disease-specific, relatively proteinase-resistant, PrP^Sc^ (Fig. 2B). The abundance of these FDC-associated PrP^Sc^ accumulations in the spleens of control-Ig treated mice had increased by 70 and 105 days after infection (Fig. 2C). Spleens from spleens anti-αL + anti-α4 treated mice also contained a similar abundance of PrP^Sc^ + FDC networks (Fig. 2A–C), suggesting that the absence of MZ B cells from the MZ at the time of IV prion injection did not impede the early accumulation of prions within the spleen.

### Temporary displacement of MZ B cells does not affect susceptibility to IV prion infection

**Figure 3:**
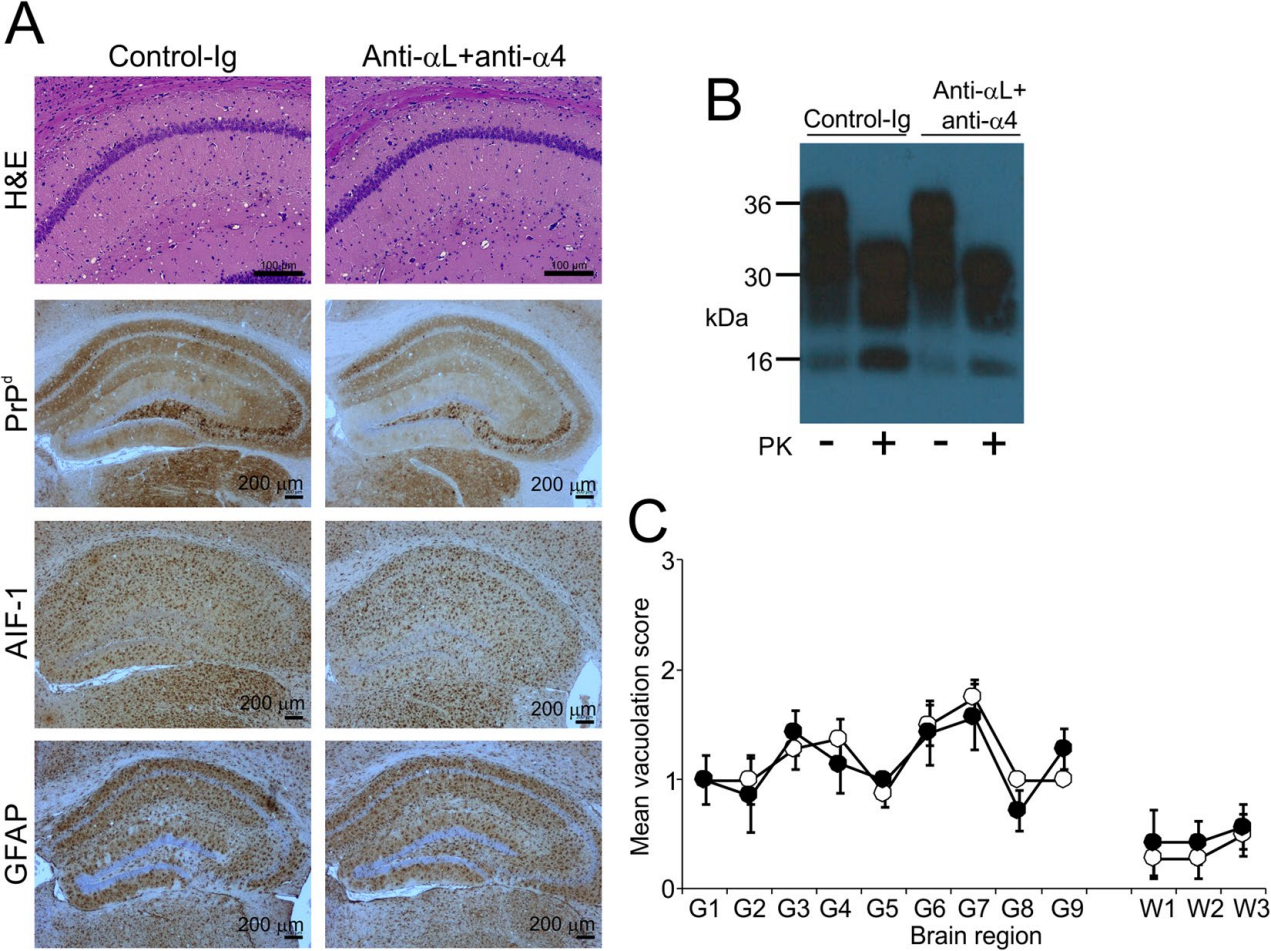
Temporary displacement of MZ B cells does not affect CNS prion disease pathogenesis. Mice were treated with anti-αL and anti-α4 mAb (anti-αL + anti-α4), or isotype-matched control-Ig, and seven days later injected IV with ME7 scrapie prions (*n* = 7-8 mice/group). Brains were collected at the terminal stage of clinical prion disease and the neuropathology compared. (**A**) Immunohistopathological analysis shows abundant spongiform pathology (H&E, upper row), PrP^d^ (brown, second row), AIF-1 + microglia (brown, third row) and GFAP + reactive astrocytes (brown, bottom row) in the brains of the clinically-affected mice from each treatment group. Haematoxylin (blue) was used as a nuclear counterstain. Scale bars, H&E panels 100 µm, all other panels 200 µm. (**B**) Immunoblot detection of high levels of prion disease-specific, relatively proteinase resistant PrP^Sc^ in representative brains of mice from each treatment group. Brain homogenates were treated in the presence ( + ) or absence (-) of proteinase K (PK) before electrophoresis. After PK treatment the samples present as a typical three-band pattern with molecular mass values between 20 to 30 kDa, representing the un-glycosylated, mono-glycosylated, and di-glycosylated isomers of PrP^Sc^ (in order of increasing molecular mass). An uncropped image of the immunoblot is provided in Supplementary Fig. S1. (**C**) The magnitude of the prion disease-specific spongiform pathology (vacuolation) was scored on a scale of 0–5 in nine distinct grey matter regions (G1–G9) and three distinct white matter regions (W1–W3): G1, dorsal medulla; G2, cerebellar cortex; G3, superior colliculus; G4, hypothalamus; G5, thalamus; G6, hippocampus; G7, septum; G8, retrosplenial and adjacent motor cortex; G9, cingulate and adjacent motor cortex; W1, inferior and middle cerebellar peduncles; W2, decussation of superior cerebellar peduncles; W3, cerebellar peduncles. Closed circles, control-Ig; open circles, anti-αL + anti-α4; *n* = 7–8 mice/group. Not significantly different to mice treated with control-Ig, Two-way ANOVA.

We next determined whether MZ B-cell displacement would affect susceptibility to IV prion infection. As above, groups of mice were treated with anti αL + anti-α4 mAb (or control-Ig) and seven days layer injected IV with a low dose of prions. All the prion injected control-Ig treated mice developed clinical prion disease with a mean survival time of 263 ± 11 days (median = 261 days, *n* = 7). Furthermore, all the prion injected anti αL + anti-α4 treated mice also developed clinical prion disease with similar survival times (mean = 269 ± 11 days, median = 266 days, *n* = 8). The characteristic neuropathological signs of prion disease including spongiform pathology (vacuolation), PrP^d^ accumulations, microglial activation (AIF-1 + cells) and reactive astrocytosis (GFAP + cells) were detected in the brains of all the clinically-affected mice from each group (Fig. 3A,B). The distribution and magnitude of the vacuolation was also similar in the brains from each treatment group (Fig. 3C). Together, our data suggest that the displacement of MZ B cells from the splenic MZ does not affect susceptibility to IV prion infection.

## Discussion

The MZ B cells express the integrins LFA-1 (αLβ2) and α4β1 highly and this enables them to be retained within the MZ through their ability to bind to the adhesion molecules ICAM-1 and VCAM-1 expressed on surfaces of the sinus lining cells^28^. When the MZ B cells are activated their expression of these integrins is down-regulated^28^, releasing them from the marginal sinus and enabling their migration into the B-cell follicle towards gradients of the chemokine CXCL13 that is synthesised by FDC and follicular stromal cells^15^. The FDC express much higher levels of complement receptors CR2/CR1 (CD21/CD35) than the surrounding B cells and this allows the FDC to strip the complement-opsonised antigens and antigen-containing immune complexes from the B cells^31^. When the MZ B cells enter the B-cell follicles their expression of the chemokine receptor CXCR5 (the receptor for CXCL13) is down-regulated, but they upregulate their expression of the sphingosine-1-phosphate (S1P) receptors^14^ S1P_1_ and S1P_3_. The blood in the marginal sinus has a relatively higher concentration of S1P when compared to the B-cell follicle, and this attracts the cells back to the MZ^14^. Since the MZ B cells continually shuttle between the MZ and B-cell follicles this provides an efficient route by which blood-borne antigens and antigen-containing immune complexes are delivered to splenic FDC^14,15^. We tested the hypothesis that MZ B cells also play mediate the initial shuttling of prions from the blood-stream to FDC. Treatment with blocking mAb against the integrins LFA-1 and α4β1 displaces the MZ B cells from the splenic marginal zone^28^, and has been used to study the role of these cells in host defence following infection with spirochete bacteria of the genus *Borrelia*^29,30^ and the intracellular parasite *Leishmania donovani*^32^. The effects of anti-αL + anti-α4 treatment on MZ B cells are transient and result in their depletion for approximately 2-3 weeks during which time the MZ is repopulated with new cells from the bone marrow^28,30^. We therefore tested the hypothesis that depletion of MZ B cells from the MZ niche by blocking integrin function^28^ would impede the initial shuttling of prions from the blood-stream to splenic FDC, and by doing so, impair IV prion disease pathogenesis. However, data in the current study demonstrate that the early accumulation of prions upon splenic FDC and susceptibility to IV prion infection were both unaltered in anti-αL + anti-α4 treated mice. This suggests that the initial propagation blood-borne prions to FDC occurs independently of MZ B cells. Of course, although the mice in this study were injected IV with a low dose of prions (20 µl of 0.1% brain homogenate), we cannot rule out the possibility that MZ B cells may play a role in prion disease pathogenesis following IV exposure to very low doses of prions.

Since the period of MZ B cells displacement in the current study was transient (approximately 2–3 weeks) it is possible that the eventual repopulation of the MZ with MZ B cells restored the propagation of the IV injected prions towards FDC. However, data from independent studies would suggest this is unlikely to be a major factor. For example, temporary blockade of tumour necrosis factor receptor 1 (TNFR1) signaling transiently de-differentiates the FDC in the spleen for approximately 1–2 weeks. Although the duration of the effects of TNFR1-blockade on FDC status were short and transient, this treatment reduced the early accumulation of prions in the spleen and significantly increased survival times after peripheral exposure^33^. In the absence of early prion accumulation upon FDC it is likely that much of the injected inoculum is engulfed and destroyed by macrophages in the spleen^11^.

Other cell populations may help mediate the initial propagation of blood-borne prions to splenic FDC. The MZ metallophilic macrophages specifically express sialoadhesin (CD169), but although prion-specific PrP^Sc^ particles in the spleens of infected mice are highly sialylated^34^, deficiency in sialoadhesin (CD169/SIGLEC1) does not affect prion accumulation upon splenic FDC or disease susceptibility^35^. The expression of SIGNR1 by MZ macrophages acts as an uptake receptor for dextran, capsular pneumococcal polysaccharides and certain complement-opsonized antigens. However, the temporary down-regulation of SIGNR1 expression in MZ macrophages also does not affect prion disease pathogenesis after IV exposure^36^. Specific populations of conventional dendritic cells (cDC; antigen-presenting bone marrow-derived mononuclear phagocytes and a distinct lineage from the stromal FDC^37,38^) are positioned within the MZ bridging channels in the spleen, and can rapidly capture blood-borne particulate antigens^39^. An absence of cDC around the time of intra-peritoneal prion exposure impedes the early accumulation of prions upon splenic FDC^40,41^. Furthermore, specific deficiency in CXCR5 expression in cDC restricts these cells from the B-cell follicles where the FDC reside and impedes the efficient propagation of prions towards FDC^42^. Thus, the cDC may instead mediate the early propagation of blood-borne prions to FDC in the spleen.

Our data do not exclude the possibility that blood-borne prions may also reach the FDC as cell-free complement-opsonised immune complexes^22–27,43^. For example, using fluorescently-labelled PrP^Sc^ fibrils Michele and colleagues suggested that small prion aggregates initially reach the draining lymph node by travelling through the afferent lymphatics in a passive cell-free manner^43^. A conduit system comprising a specialised network of stromal cells has been identified in the spleen that enables small blood-borne molecules (approx. ≤70 kDa) to be distributed throughout the white pulp^44^. A specific conduit system has also been described that forms a physical connection between the MZ and FDC within the B cell follicles^45^. Whether blood-borne monomers or dimers of prion-specific PrP^Sc^ are also passively delivered to FDC via the splenic conduit system or other route in sufficient magnitude to establish host infection remains to be determined.

The integrins αLβ2 (LFA-1) and α4β1 may also be expressed by other immune cell subsets. However, our IHC analysis suggested that αL + anti-α4 mAb treatment did not affect the abundance and distribution of follicular B cells (IgD-hi cells), MZ macrophages and marginal metallophilic macrophages in the spleen. Although this treatment can affect T cell-activation^30^, peripheral prion disease pathogenesis is not affected in mice deficient in T cells^46–48^.

The susceptibility of aged mice (~600 days old) to peripheral prion infection is dramatically reduced in contrast to young mice (~42–56 days old) and coincides with ageing-related disturbances to the microarchitecture of the secondary lymphoid tissues that impede the ability of FDC to efficiently acquire and replicate prions^12,49–51^. Specifically in the aged spleen the positioning and density of marginal sinus lining cells, MZ macrophages, MZ metallophilic macrophages and MZ B cells are each grossly disturbed^12,13,49,52,53^. As a consequence of these ageing-related disturbances to the MZ the propagation of blood-borne antigen-containing immune complexes and prions to FDC is adversely affected^12,13^. However, in the current study we show MZ B cell depletion did not affect the early accumulation of prions upon FDC. This suggest that the effects of ageing on prion accumulation upon FDC^12^ were also unlikely to be due to the effects of ageing on MZ B cells^13^. The expression of cellular PrP^C^ in FDC is essential for them to be able to replicate prions^11^. Since PrP^C^ expression in FDC is substantially reduced in aged mice this will significantly impact on their ability to replicate prions^12,49^. Whether ageing-related disturbances and thickening of the MZ^13^ adversely affect the blood flow through marginal sinus, reducing the shuttling of immune-complexes and prions to FDC, remains to be determined.

Further studies are necessary to determine how blood-borne prions establish infection upon splenic FDC. Treatments that impede the initial amplification of prions upon FDC can reduce susceptibility to peripherally-acquired prion infections^9,10,22,33,54^. Thus, characterisation of the cellular and molecular mechanisms that prions utilise to establish infection within the spleen may reveal novel prophylactic or therapeutic targets for these currently untreatable and devastating diseases.

## Materials and Methods

### Ethics statement

Ethical approvals for the *in vivo* mouse experiments were obtained from The Roslin Institute’s and University of Edinburgh’s ethics committees. All the experiments in this study were undertaken in accordance with the guidelines and regulations of the UK Home Office ‘Animals (scientific procedures) Act 1986’ and were performed under the authority of UK Home Office Project Licence PPL60/4325. Appropriate care was given to reduce harm and suffering, with anaesthesia was administered where necessary. At the end of the experiments the mice were humanely culled by cervical dislocation.

### Mice

Female C57BL/6 J mice were obtained from Charles River Laboratories (Charles River, Margate, UK) and housed under specific pathogen-free conditions with a 12:12 h light:dark cycle. Food and water were provided *ad libitum*. Mice were used in studies at 6–8 weeks old.

### *In vivo* anti-integrin antibody treatment

Transient displacement of MZ B cells was achieved by IV injection with 100 µg each of rat anti-mouse LFA-1 mAb (CD11a, clone M17/4, IgG2aκ) and rat anti-mouse integrin α4 mAb (CD49d, clone R1-2, IgG2bκ) as described previously^28–30^. Where indicated some mice were injected with non-specific rat IgG2aκ (clone eBR2a) and rat IgG2bκ (clone eB149/10H5) as isotype controls. All these antibodies were purchased from ThermoFisher (Loughborough, UK).

### Flow cytometry

Single spleen cell suspensions were prepared and red blood cells lysed using red blood cell lysis buffer (Sigma, Poole, UK). Viable cells were counted and re-suspended in FACS buffer (PBS pH 7.4 containing 0.1% BSA, 0.1% sodium azide and 0.02% EDTA). Non-specific immunoglobulin-binding was blocked using Mouse Seroblock FcR (Bio-Rad Laboratories Watford, UK) and cells subsequently immunostained with the following mAb purchased from BioLegend (London, UK): anti-mouse CD1d-PerCP/Cy5.5 (clone Ly-38); anti-mouse CD21/35-Pacific Blue (clone 7G6); anti-mouse CD45R:B220-APC (clone RA3-6B2). Relevant non-specific antibody isotypes were used as controls. Cells were analysed on a LSR Fortessa with DIVA software (BD Biosciences). Cells were gated on lymphocytes, doublets excluded and data analysed with FlowJo software (FlowJo, LLC, Ashland OR, USA).

### Intravenous prion infection

Mice were injected IV with 20 µl of a 0.1% (weight/volume) brain homogenate prepared from mice terminally infected with ME7 scrapie prions (containing approximately 1 × 10^3^ ID_50_ units). The mice were then coded, and assessed blindly for the clinical signs of prion disease by independent husbandry technicians. Mice were culled at a standard clinical endpoint as described^55^. The clinical status of each mouse was confirmed by histopathological assessment of the prion disease-specific spongiform vacuolation in haematoxylin and eosin stained brain sections as described^56^.

### Immunohistochemistry

Snap-frozen spleens were embedded in optimal cryotomy temperature compound and cryosectioned at 10 μm thickness. Sections were then immunostained using the following antibodies: rat anti-mouse CD1d (clone 1B1; Bio-Rad Laboratories); rat anti-mouse CD21/35 (clone 7G6; BD Biosciences); anti-mouse CD45R:B220 (clone RA3-6B2); anti-mouse CD169 (MOMA-1; Bio-Rad Laboratories); Alexa Fluor 488-conjugated anti-mouse IgD (clone 11–26 c.2a; Biolegend); Alexa Fluor 594-conjugated goat anti-mouse IgM (µ chain; ThermoFisher); anti-mouse MARCO (clone ED31; Bio-Rad Laboratories); Armenian hamster anti-mouse SIGNR1 (clone 22D1; eBioscience). Where appropriate, binding of primary antibodies was detected using biotin- or fluorophore-conjugated goat anti-species specific secondary antibodies (Jackson Immunoresearch, West Grove, PA). The binding of biotinylated secondary antibodies was visualized using the Elite ABC/HRP kit (Vector Laboratories, Peterborough, UK) with diaminobenzidine (DAB) or NovaRed (Vector Laboratories) as substrates.

Spleens and brains from mice infected with prions were fixed in periodate-lysine-paraformaldehyde, processed on an ASP300S automated tissue processor (Leica), embedded in paraffin wax and 5 µm sections prepared. Detection of disease-specific PrP (PrP^d^) was enhanced by hydrated autoclaving (15 min, 121 °C, hydration) followed by immersion in formic acid (98%) for 5 min. PrP-specific polyclonal antiserum 1B3^57^ was then used to detect PrP. Anti-glial fibrillary acidic protein (GFAP; DAKO, Ely, UK) was used to detect astrocytes. For the detection of microglia the sections were treated with Target Retrieval Solution (DAKO) and immunostained using anti-AIF-1/Iba1 (Wako Chemicals GmbH, Neuss, Germany). Appropriate species-specific biotin-conjugated secondary antibodies (Jackson ImmunoResearch Europe Ltd., Ely, UK) were subsequently used followed by HRP-conjugated to the avidin-biotin complex (ABC kit, Vector Laboratories, Peterborough, UK), and immunolabelling detected using diaminobenzidine (DAB; Sigma). Paraffin-embedded tissue immunoblot analysis was used to discriminate between prion disease-specific proteinase K-resistant PrP^Sc^ and cellular PrP^C^ as described^58^.

For light microscopy, haematoxylin was used as a counterstain and sections imaged on a Ni.1 microscope (Nikon). For immunofluoresce the immunostained sections were mounted in fluorescent mounting medium (DakoCytomation, Glostrup, Denmark) and imaged using a Zeiss LSM710 confocal microscope with ZEN software (Zeiss).

### Western Immunoblot analysis

Brains samples were homogenised in NP-40 lysis buffer (1% NP-40, 0.5% sodium deoxycholate, 150 mM NaCl, 50 mM Tris-HCL [pH 7.5]) at 10% weight/volume, and subsequently treated at 37 °C for 1 h with proteinase K (20 µg/ml). Samples were then separated by electrophoresis and electroblotted onto polyvinylidene difluoride membranes as described previously^42^. PrP was detected using anti-mouse PrP mAb (clone 7A12; Yin *et al*., 2007), followed by HRP-conjugated anti-mouse antibody (Jackson Immunoresearch, Ely, UK) and visualised with BM Chemiluminescent substrate kit (Roche, Burgess Hill, UK). An uncropped image of the immunoblot is provided in Supplementary Fig. S1.

### Statistical Analyses

Data are presented as mean ± SEM. Significant differences between groups were determined by Student’s *t*-test, unless indicated otherwise otherwise. Values of *P* < 0.05 were accepted as significant.

## Supplementary information

Supplementary Figure S1

## Data Availability

All relevant data are presented within the paper.
