# Supplementary material for: Unaltered intravenous prion disease pathogenesis in the temporary absence of marginal zone B cells
Source: Sci Rep. 2019 Dec 13;9:19119. doi: 10.1038/s41598-019-55772-w (PMC6910919; doi:10.1038/s41598-019-55772-w)

Unaltered intravenous prion disease pathogenesis in the temporary absence of marginal zone B cells

Barry M. Bradford[1] & Neil A. Mabbott[1]

[1] The Roslin Institute & Royal (Dick) School of Veterinary Sciences, University of Edinburgh, Easter Bush EH25 9RG, United Kingdom

Supplementary information

Supplementary Figure S1. A full image of the uncropped immunoblot used in Figure 3B.

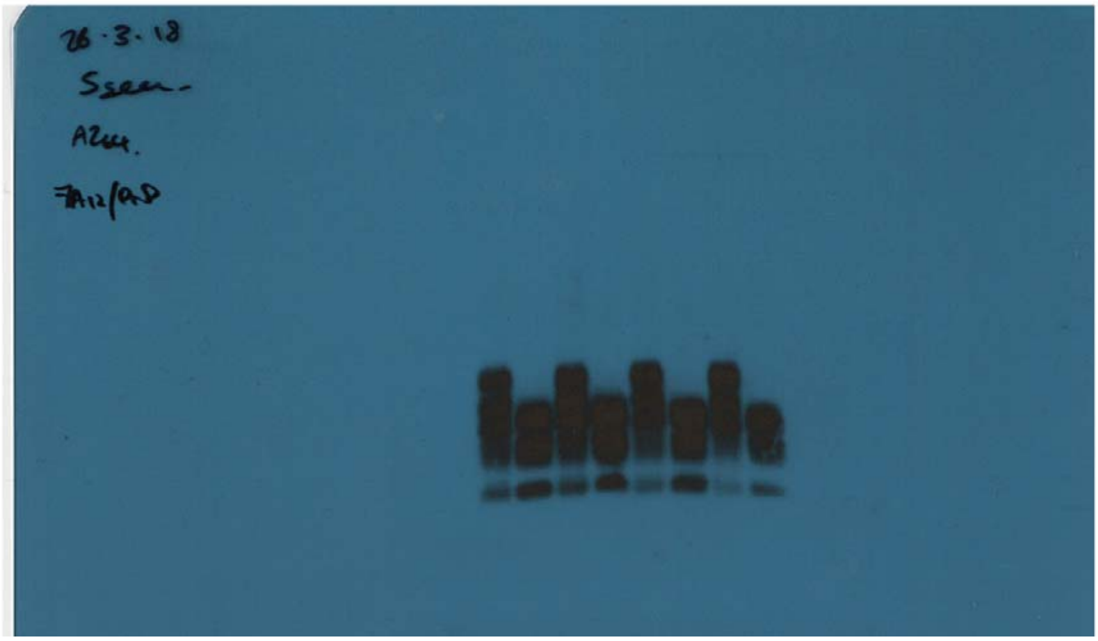

Supplement: Supplementary file 1 — Supplementary Figure S1 [file 41598_2019_55772_MOESM1_ESM.pdf]
